# Homeologous regulation of Frigida-like genes provides insights on reproductive development and somatic embryogenesis in the allotetraploid *Coffea arabica*

**DOI:** 10.1038/s41598-019-44666-6

**Published:** 2019-06-11

**Authors:** Natalia Gomes Vieira, Ilse Fernanda Ferrari, Juliana Costa de Rezende, Juliana Lischka Sampaio Mayer, Jorge Maurício Costa Mondego

**Affiliations:** 1 Instituto Agronômico de Campinas, IAC, Centro de Pesquisa e Desenvolvimento em Recursos Genéticos Vegetais, Campinas, 13075-630 Brazil; 2 Universidade Estadual de Campinas, UNICAMP, Programa de Pós-graduação em Genética e Biologia Molecular, Campinas, 13083-970 Brazil; 3 Empresa de Pesquisa Agropecuária de Minas Gerais, Empresa de Pesquisa Agropecuária de Minas Gerais, EPAMIG Sul, Lavras, 37200-000 Brazil; 4 Universidade Estadual de Campinas, UNICAMP, Departamento de Biologia Vegetal, Instituto de Biologia,, Campinas, 13083-862 Brazil

**Keywords:** Transcription, Plant molecular biology

## Abstract

*Coffea arabica* is an allotetraploid of high economic importance. *C*. *arabica* transcriptome is a combination of the transcripts of two parental genomes (*C*. *eugenioides* and *C*. *canephora*) that gave rise to the homeologous genes of the species. Previous studies have reported the transcriptional dynamics of *C*. *arabica*. In these reports, the ancestry of homeologous genes was identified and the overall regulation of homeologous differential expression (HDE) was explored. One of these genes is part of the *FRIGIDA*-like family (*FRL*), which includes the *Arabidopsis thaliana* flowering-time regulation protein, *FRIGIDA* (*FRI*). As nonfunctional *FRI* proteins give rise to rapid-cycling summer annual ecotypes instead of vernalization-responsive winter-annuals, allelic variation in *FRI* can modulate flowering time in *A*. *thaliana*. Using bioinformatics, genomic analysis, and the evaluation of gene expression of homeologs, we characterized the *FRL* gene family in *C*. *arabica*. Our findings indicate that *C*. *arabica* expresses 10 *FRL* homeologs, and that, throughout flower and fruit development, these genes are differentially transcribed. Strikingly, in addition to confirming the expression of *FRL* genes during zygotic embryogenesis, we detected *FRL* expression during direct somatic embryogenesis, a novel finding regarding the *FRL* gene family. The HDE profile of *FRL* genes suggests an intertwined homeologous gene regulation. Furthermore, we observed that *FLC* gene of *C*. *arabica* has an expression profile similar to that of *CaFRL* genes.

## Introduction

*Coffea arabica* and *C*. *canephora* are the species responsible for the production of all coffee beans worldwide. As an allotetraploid (2n = 4x = 44), the *C*. *arabica* genome is composed of the diploid genomes (2n = 2x = 22) of its ancestors, *C*. *canephora* and *C*. *eugenioides*, which became subgenomes within this species (CaCc and CaCe, respectively)^1–3^. *Coffea eugenioides* is a bush-like plant that inhabits mild-temperature highlands and produces low caffeine-containing small fruits^4^. *Coffea canephora* trees inhabit warm tropical-equatorial lowlands and produce high caffeine-containing seeds^5^. The two parental species are closely related, and the two subgenomes in *C*. *arabica* have low sequence divergence (i.e., 1.3% average difference in the genes)^1^, which is also correlated with the autogamous reproductive strategy of *C*. *arabica*.

Several studies have found that the transcriptional set of *C*. *arabica* is a combination of the homeologous gene expression of the CaCc and CaCe subgenomes^3,6–12^. It is extremely likely that the homeologous differential expression (HDE) in *C*. *arabica* is responsible for the plasticity in phenotype modulation in different tissues and under different biological conditions. In fact, allopolyploidization has been considered a contributor to speciation and plant adaptation to broader habitats^6,13–16^. Although homeolog loss and silencing were found to be common in the CaCc subgenome, which suggested CaCe dominance, neither of the two subgenomes were preferentially expressed in *C*. *arabica*^8^. Therefore, it appears that each gene has its own homeologous expression coordination, providing global intertwined homeolog regulation in *C*. *arabica*.

In *Arabidopsis thaliana*, FRIGIDA (FRI) is a key protein that regulates flowering transition by activating the *flowering locus C* (*FLC*), which encodes a central flowering repressor that controls the plant response to vernalization^17–19^. FRI acts as a scaffold protein that interacts with other proteins to assemble a complex that binds to the *FLC* promoter region, thereby triggering its expression, and consequently, inhibiting flowering^20^. On the contrary, vernalization has no effect on *FRI* expression, and instead promotes flowering by causing the epigenetic repression of *FLC* expression^19^.

*FRL*s (FRIGIDA-Like genes) have been found in all sequenced plant genomes, regardless of whether the species displays vernalization. Even though *FRI* is connected to flowering regulation, members of this gene family are also associated with other biological processes connected with reproduction, such as embryonic development^21^ and seed maturation^22^. Based on single-nucleotide polymorphism (SNP)–based detection of homeologous genes, two *C*. *arabica* FRLs were suggested to display HDE^3^ (more details in the Methods section). Given the advances in genome and transcriptome sequencing of *C*. *arabica*, *C*. *canephora*, and *C*. *eugenioides*^23–25^, we further characterized the *Coffea FRL*s by evaluating their sequence features, phylogenetics, and *cis*-regulatory elements, and by characterizing the *C*. *arabica FRL* transcription and HDE in tissues such as flowers and fruits, and during direct somatic embryogenesis. In addition, *C*. *arabica FLC* expression was evaluated, which indicated an expression profile similar to that of *FRL* genes. Our results provide strong support to the hypothesis that *FRL*s are active in diverse stages of plant reproduction.

## Results

### Characterization of *FRL* genes in *Coffea*

Eight sequences of *A*. *thaliana FRL*s were used to search the BlastP database against the *C*. *canephora* genome sequence^23^. Five genes were found to be similar to the corresponding genes in *A*. *thaliana* (Table 1). Next, *C*. *canephora FRL* sequences were used in the BlastP search against the *C*. *arabica* genome sequence (http://www.phvtozome.net) and the *C*. *eugenioides* EST databank^25^. Five sequences were found in *C*. *eugenioides* and 10 in *C*. *arabica*. After aligning all the *FRL*s from the abovementioned *Coffea* species, it was possible to assign *C*. *arabica* homeologous *FRL*s using the same SNP alignment–based strategy^3^. Genes considered as present in *C*. *canephora* subgenome were designated as x.1, and the genes considered as present in *C*. *eugenioides* subgenome were designated as x.2 (CaCe; Table 1). It should be mentioned that, because we did not have access to the *C*. *eugenioides* genome yet, the *C*. *eugenioides FRL* genes were not completely described (e.g., complete gene annotation, presence of genes, and orthologs).

**Table 1:** FRI-related genes in coffee. CDS size (CDSs), number of introns (I), protein length (aa), similarity (S).

Gene ID	Localization ID	Ncbi ID	CDSs (bp)	I	aa	At orthologs (Gene ID)	S
*CcFRL-1*	Cc01_g15840	CDP03992	1860	4	619	AtFRL3 (AT5G483851)	72%
*CaFRL-1*.*1*	Scaffold_2016.624		1644	2	547		72,6%
*CaFRL-1*.*2*	Scaffold_635.49		1869	4	622		71,5%
*CcFRL-2*	Cc03_g03790	CDO99060.1	1596	2	532	AtFRL4a (AT3G224401) AtFRL4b (AT4G149001)	80% 81%
*CaFRL-2*.*1*	Scaffold_315.439		1599	2	532		73% 75,9%
*CaFRL-2*.*2*	Scaffold_624.657		1593	2	530		73,2% 76,3%
*CcFRL-3*	Cc04_g05540	CDO98273.1	2046	3	681	AtFRI (AT4G006501)	59%
*CaFRL-3*.*1*	Scaffold_352.665		1530	2	509		71,6%
*CaFRL-3*.*2*	Scaffold_633.267		1530	2	509		71,1%
*CcFRL-4*	Cc05_g14640	CDP13747.1	832	2	519	AtFRL1 (AT5G163201) AtFRL2 (AT1G318141)	53% 52%
*CaFRL-4*.*1*	Scaffold_770.1281		1560	2	519		52,8% 50,7%
*CaFRL-4*.*2*	Scaffold_770.842		1338	3	445		46,9% 50%
*CcFRL-5*	Cc00_g14390	CDP19997.1	2307	3	768	AtFrigida-like (ATG272201)	45%
*CaFRL-5*.*1*	Scaffold_632.618		2169	2	722		45,3%
*CaFRL-5*.*2*	Scaffold_2286.135		2322	2	773		45,3%

*Arabdopsis thaliana* (At), base pairs (bp).

**Figure 1:**
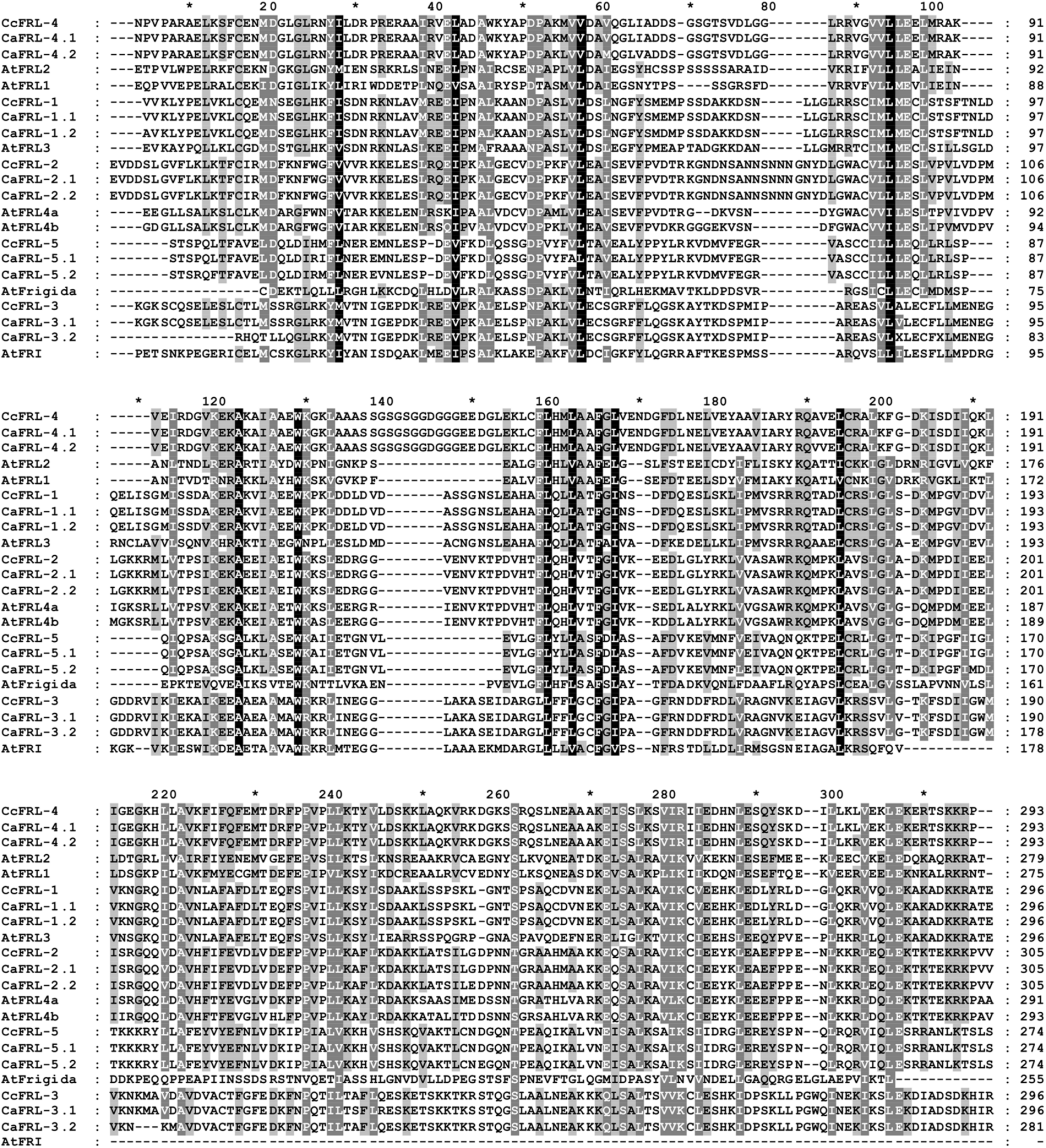
Sequence alignment of Frigida domain in *Coffea* (Ca, Cc) and *Arabdopsis* (At) Frigida-like proteins. Black background, more than 90% of conservation between amino acids; Dark gray background and white letters, conservation between amino acids 89–80%; Light gray background and black letters, conservation between amino acids 79–60%.

FRIGIDA domain PF07899 (https://pfam.xfam.org) was detected in all the analyzed sequences as well as those used for protein alignment (Fig. 1). In addition, the N-terminus of the FRLs was evaluated to classify *Coffea* FRLs within gene families based on protein sequence analyses described by Risk *et al*.^22^. Supplementary Fig. S1 (supplementary note) shows the five FRL families and the presence of members of *C*. *canephora* and *C*. *arabica* in each *A*. *thaliana* FRL family.

**Figure 2:**
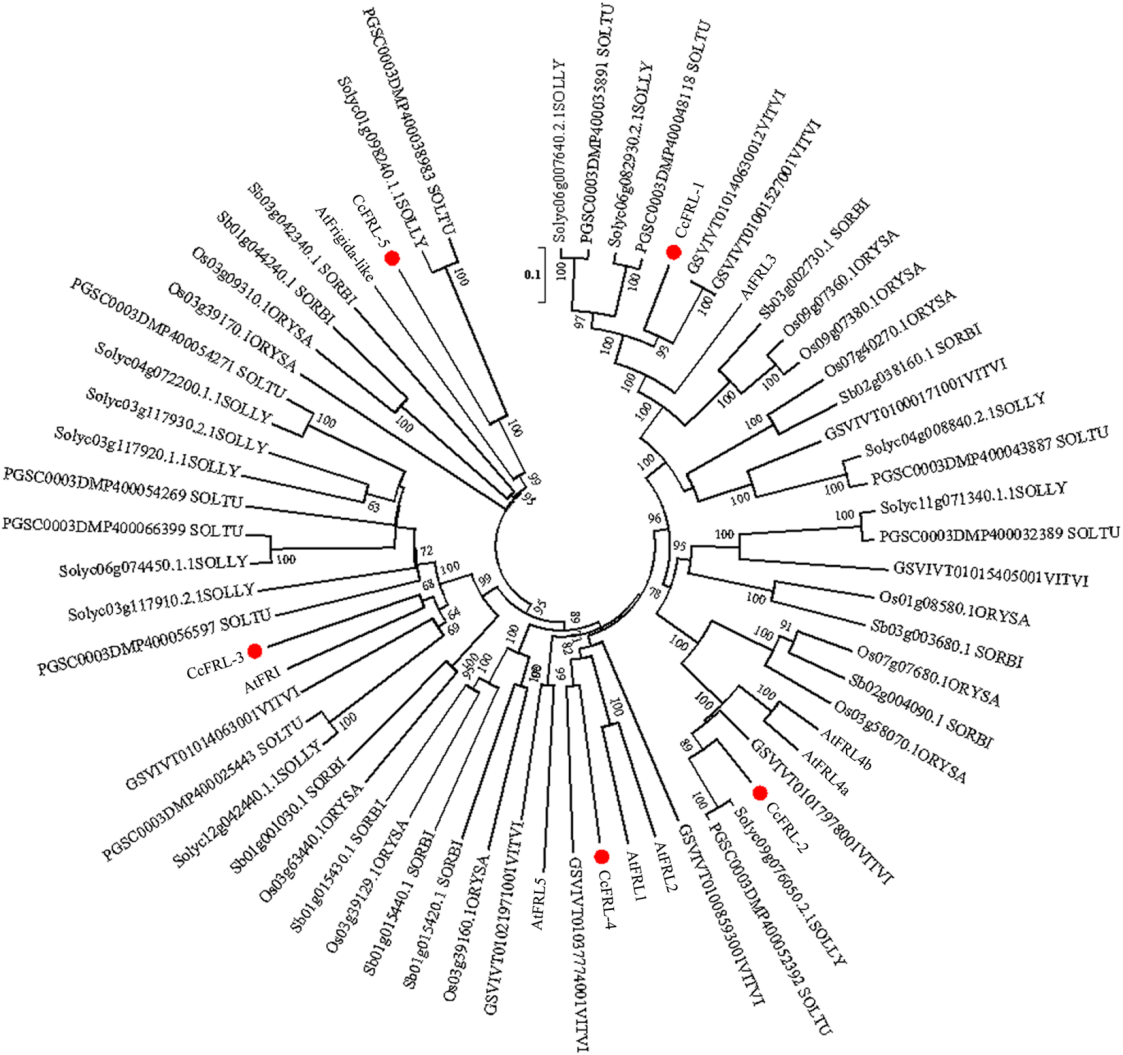
Phylogenetic analysis of CcFRL proteins with orthologous proteins of *A*. *thaliana* (At), *S*. *bicolor* (Sorbi), *O*. *sativa* (ORYSA), *S*. *lycopersicum* (SOLLY), *S*. *tuberosum* (SOLTU) and *V*. *vinifera* (VITVI). The percentage of replicate trees in which the associated taxa clustered together in the bootstrap test (1000 replicates) is shown next to the branches. Bootstrap values >50 are shown on the tree. The evolutionary distances were computed using the p-distance method.

To gain insight into the evolutionary relationships of *FRL* genes in *Coffea* and other plant genomes, the neighbor-joining method was used to construct a phylogenetic tree (Fig. 2). Sequences of *C*. *canephora* FRL and its respective *A*. *thaliana* orthologs were grouped within the same clade (Fig. 2). Mixed sequences from monocotyledons and dicotyledons were found within different clades, suggesting an ancestral *FRL* origin before the divergence of the plant clades. In addition, we observed that each *C*. *canephora* FRL was allocated to an FRL subfamily, as described by Risk *et al*.^22^, which is not the case for tomato and potato (Solanaceae), which lack AtFRL1 homeologs, and rice and sorghum (Poaceae), which lack AtFRI homeologs (Fig. 2).

### *Coffea arabica* homeologous *FRL* assignments

**Figure 3:**
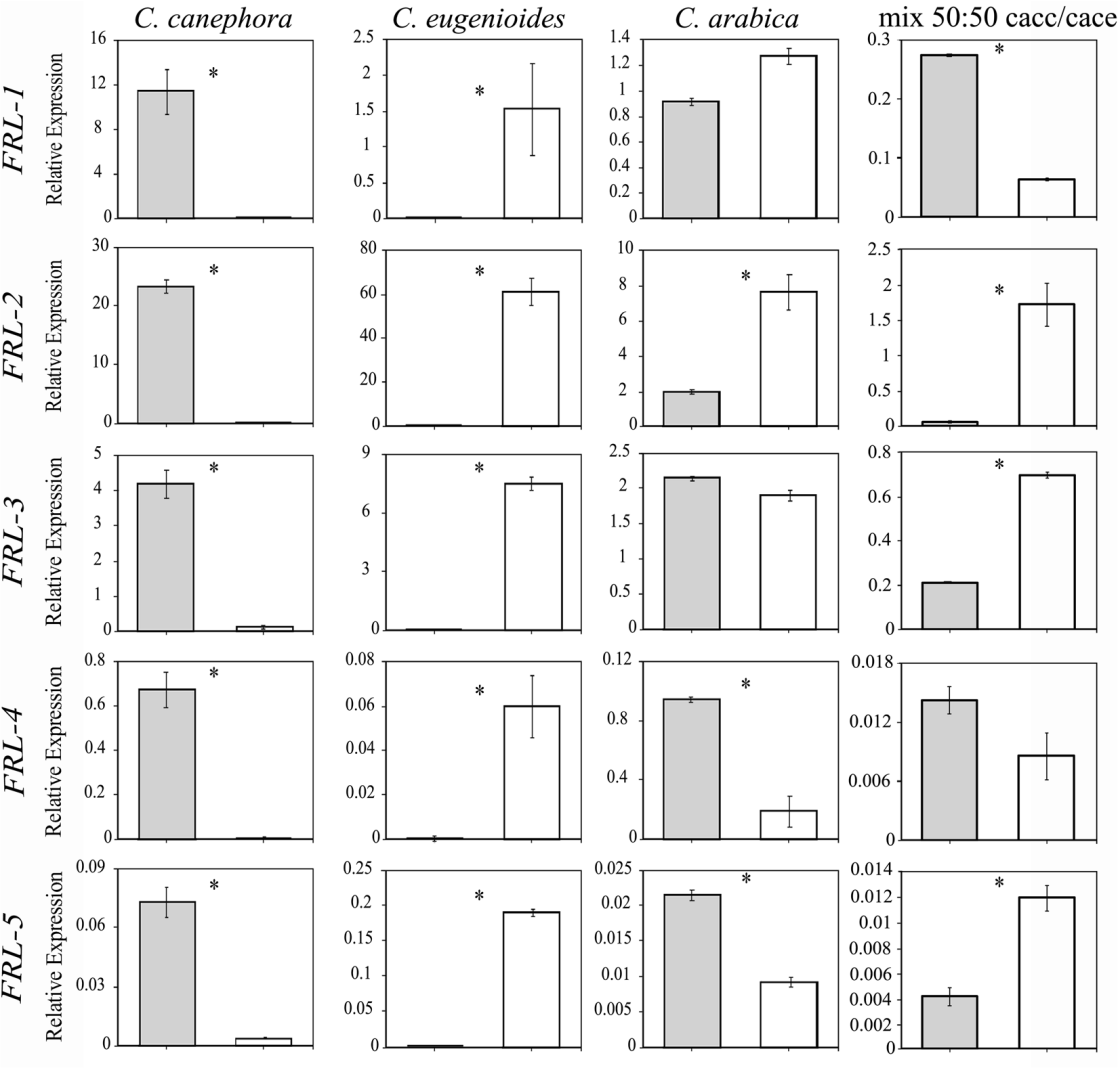
Expression profiles of *CaFRL* homeologous genes (CaCc and CaCe) in leaves of *C*. *arabica*, *C*. *canephora*, *C*. *eugenioides* and a 50:50 mix of the parental cDNAs (*C*. *canephora* and *C*. *eugenioides*). Gray bars refer to CaCc and white bars refer to CaCe. Values of three technical replicates are presented as mean ± SD (error bars). Transcript abundances were normalized using the expression of *UBI* (ubiquitin) as reference gene. Asterisks indicate significant differences (P < 0.05) between homeologous genes.

SNP alignment-based strategy was used to assign the *FRL* homeologous genes in *C*. *arabica*. Briefly, *C*. *arabica* (2), *C*. *canephora* (1), and *C*. *eugenioides* (1) sequences similar to each *A*. *thaliana FRL* were aligned based on the SNP profile. *C*. *arabica* genes were assigned as derived from the *C*. *canephora* subgenome (CaCc; *FRL* x.1) or *C*. *eugenioides* subgenome (CaCe; *FRL* x.2). The *C*. *arabica*, *C*. *eugenioides*, and *C*. *canephora FRL* genes were aligned to construct a dendogram, which confirmed the subgenome assignment (Supplementary Fig. S2, supplementary note). SNPs observed *in silico* allowed for designing of a homeolog-specific primer in *C*. *arabica* according to the TaqMAMA method^26^ (Supplementary Table S1, supplementary note), or containing an indel of at least three nucleotides (Supplementary Fig. S3, supplementary note). The subgenome specificity of each homeolog-specific primer was tested with quantitative real-time (qRT-PCR) using cDNA from the leaves of *C*. *canephora*, *C*. *eugenioides*, and *C*. *arabica*. As expected, the primers designed from the CaCe subgenome amplified only the *C*. *eugenioides* cDNA, and primers that matched the CaCc subgenome amplified only the *C*. *canephora* cDNA (Fig. 3, left and middle columns). In contrast, both the primers were effective in amplifying the *C*. *arabica* homeologs (CaCe and CaCc) in each *FRL*, indicating that these genes and, consequently, both the subgenomes, are transcriptionally active in *C*. *arabica* (Fig. 3, right column).

Interestingly, the *FRL* homeologs displayed different expression profiles in *C*. *arabica* leaves. For example, *CaFRL-1* and *CaFRL-2* CaCe homeologs (*FRL* x.2) were expressed more than were the CaCc homeologs (*FRL* x.1). Inversely, for *CaFRL-4* and *CaFRL-5*, the homeologous expression of CaCc was greater than that of CaCe (Fig. 3). The *CaFRL-3* homeologous expression was balanced (Fig. 3).

To further verify the homeolog-specific findings, qPCR experiments using a 50:50 mix of the parental cDNAs were performed to ensure that the primers, when being amplified from the tetraploid, were indeed behaving in a homeolog-specific manner. Briefly, we made a 50:50 mix of cDNA from *C*. *eugenioides* and *C*. *canephora*, and carried out qPCR with homeolog-specific primers for each gene. We did not perform multiplex analysis but included the cDNA mix and each homeolog primer in separate wells. We confirmed the amplification of each homeolog in the mix, and interestingly, noted a similar expression rate as seen in ancestral samples, when comparing the expression scales of ancestral amplifications with that of the 50:50 mix (Fig. 3).

The homeologous promoters of *CaFRL*s were also assigned and evaluated with an aim to find the putative differential *cis*-elements among them. The results can be found in the Supplementary file (Figs S4–S7, supplementary note).

### *Coffea arabica FRL* expression during flower development

**Figure 4:**
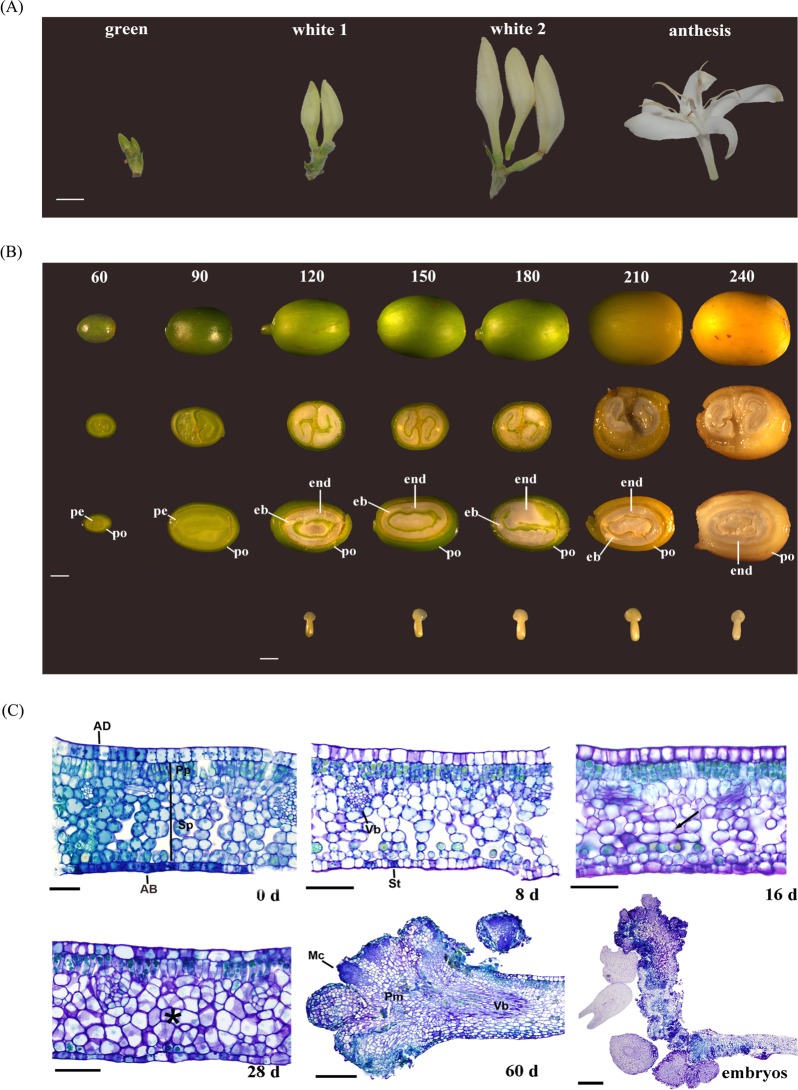
Anatomical view of *C*. *arabica* organs and tissues at which *CaFRL* gene expression was evaluated. (**A**) Flowers at different stages (green cluster, white cluster, white candle and anthesis). Scale: 5 mm. (**B**) Fruits (From top to bottom: whole fruit, fruit cross section, fruit longitudinal section, embryos; from left to right: days after flowering). Perisperm (pe), embryo (eb), endosperm (end), pericarp (pe). Scale: fruits = 2 mm, embryos = 1 mm. (**C**) Foliar explants collected throughout DSE. 0 days (0d), 8 days (8d), 16 days (16d), 28 days (28d), 60 days (60d), embryo formation (Embryos). Adaxial epidermis (AD), palisade parenchyma (Pp), spongy parenchyma (Sp),vascular bundle (Vb), abaxial epidermis (AB), stomata (St); asterisk indicates intense mitosis in spongy parenchyma; Arrow indicates the beginning of cellular division at spongy parenchyma; Pró-embryonic mass (Pm); Meristematic cells (Mc) Scale: 0d-28d = 50 µm, 60d and Embryos = 200 µm.

**Figure 5:**
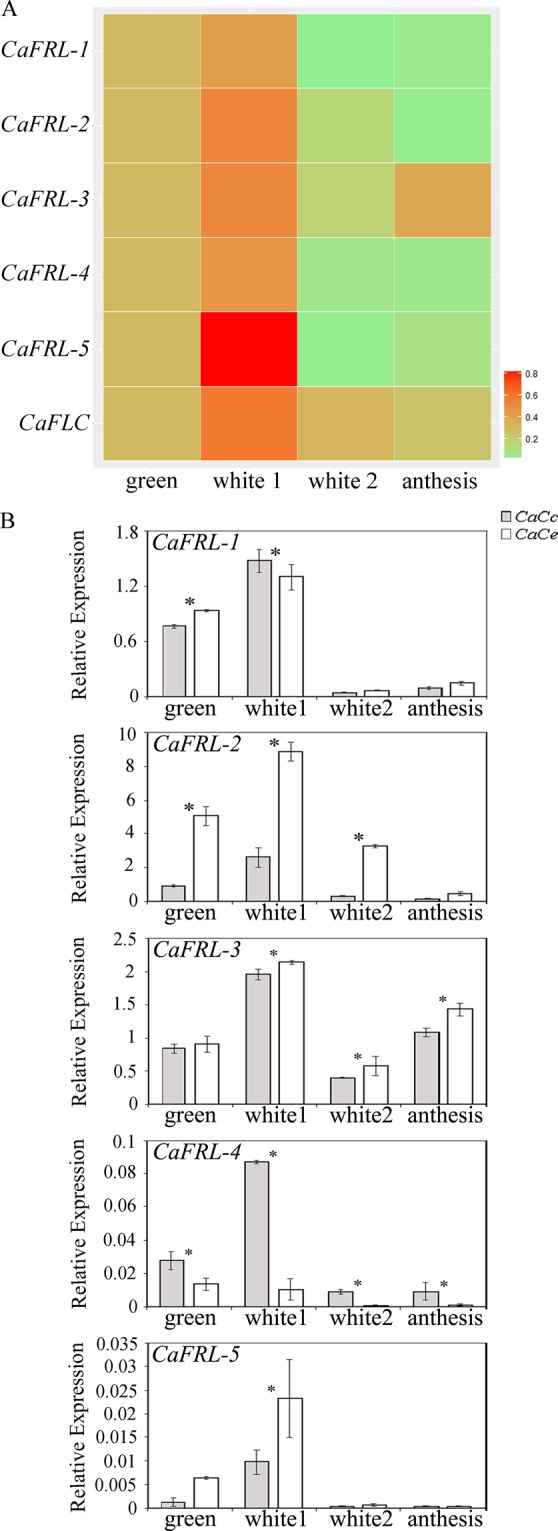
Gene expression analysis of *CaFRL* genes and *CaFLC* gene in *C*. *arabica* flowers. (**A**) Heat map visualization of *CaFRL* expression in flowers at different stages (see material and methods). The sum of relative homeolog expressions was used as numerical input for creating the heat map scale from light green (weakly expressed) to red (strongly expressed). ‘Green cluster’ sample was used as internal calibrator. (**B**) Expression profiles of homeologous genes (CaCc and CaCe) of *CaFRL* family in flowers at different stages (green cluster, white 1 floral bud, white 2 floral bud and anthesis). Values of three technical replicates are presented as mean ± SD (error bars). Transcript abundances were normalized using the expression of *UBI* (ubiquitin) as reference gene. Asterisks indicate significant differences (P < 0.05) between homeologous genes.

*CaFRL* expression was assessed during four stages of *C*. *arabica* floral development, depicted in Fig. 4A: green floral buds, white floral buds <10 mm (white 1), white floral buds >10 mm (white 2), and open flowers (anthesis). All the *CaFRLs* showed higher expression levels in the white 1 stage than in other floral stages, and in particular, *CaFRL-5* (Fig. 5A) showed a decay in transcription during advanced floral development. The evaluation of HDE (Fig. 5B) revealed that the expressions of *CaFRL-2*, *CaFRL-4*, and *CaFRL-5* were subgenome biased (*CaFRL-2* and *CaFRL-5* toward CaCe and *CaFRL-4* toward CaCc), whereas *CaFRL-1* and *CaFRL-3* homeologs tended to be similarly expressed throughout flower development (Fig. 5B).

### *Coffea arabica FRL* expression during fruit development

Coffee fruit development is a long process that can be evaluated using the cross and longitudinal sections of the fruit. Between 60 and 90 DAF, the perisperm (inner fruit) and pericarp (outer fruit) develop. Perisperm, a prevalent inner tissue, gradually disappears and is replaced by the endosperm, surrounded by a thin tissue of silver skin membrane (Fig. 4B). By 120 DAF, the embryo can be visualized, and by 180 DAF, it achieves its final length and morphology (Fig. 4B). Based on these macroscopic parameters, 60 and 90 DAF contained perisperm and pericarp samples, while the subsequent harvest days contained pericarp, endosperm, and embryo (Fig. 4B).

**Figure 6:**
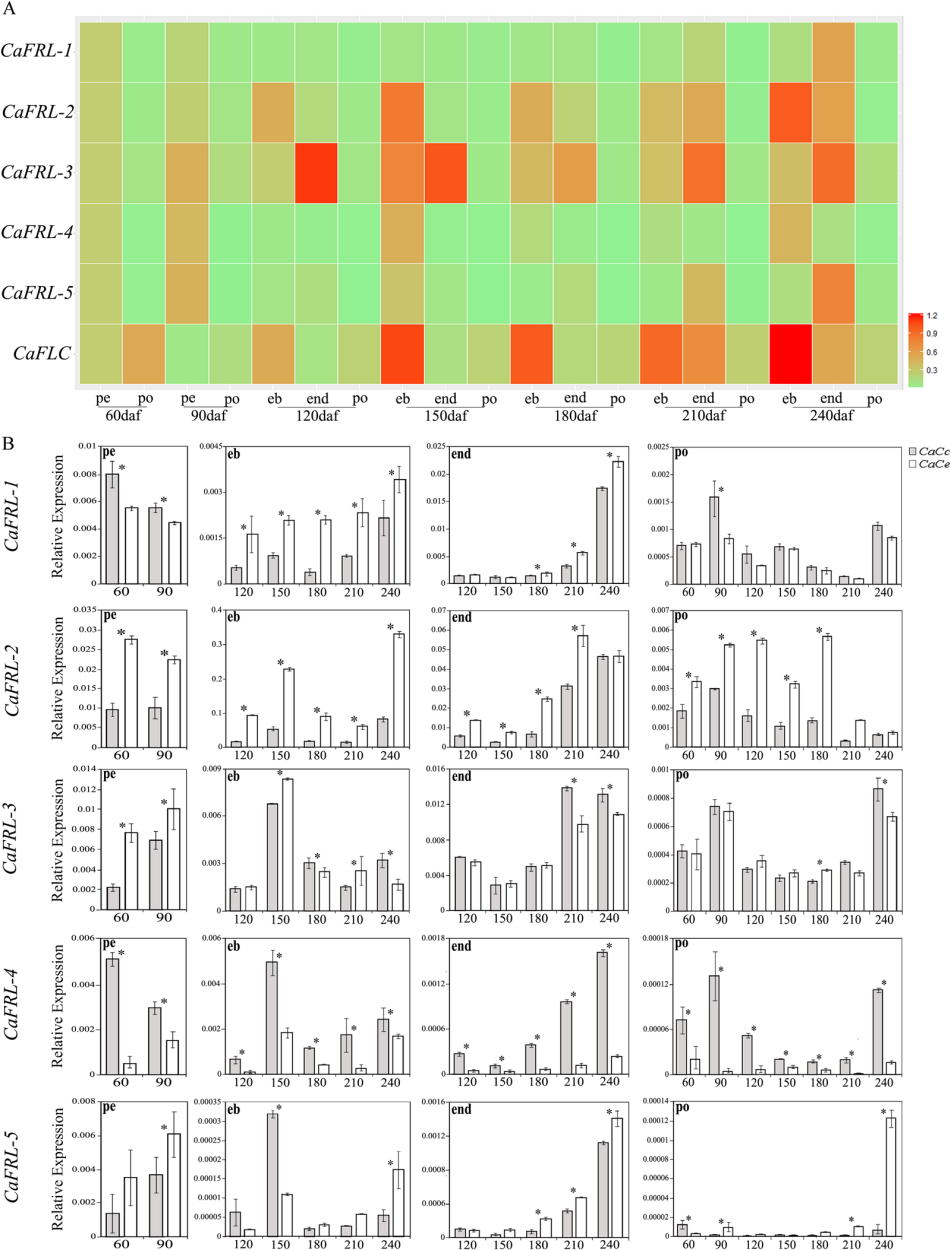
Gene expression analysis of *CaFRL* genes and *CaFLC* gene during *C*. *arabica* fruit development (**A**) Heat map visualization of *CaFRL* expression in fruits at different stages of fruit development. The sum of relative homeolog expressions was used as numerical input for creating the heat map scale, from light green (weakly expressed) to red (strongly expressed). ‘60 daf pe’ sample was used as internal calibrator (**B**) Expression profiles of homeologous genes (CaCc and CaCe) of *CaFRL* family in fruits at different tissues. Perisperm (pe), embryo (eb), endosperm (end) and pericarp (po) and stages of ripening (60–240 daf, days after flowering). Values of three technical replicates are presented as mean ± SD (error bars). Transcript abundances were normalized using the expression of *UBI* (ubiquitin) as reference gene. Asterisks indicate significant differences (P < 0.05) between homeologous genes.

The expression analysis heat map showed that all *CaFRL*s were expressed in fruits, especially in the embryo and endosperm, with different transcriptional profiles (Fig. 6). *CaFRL-1* had a nearly specific expression that manifests during the late-endosperm stage (Fig. 6A). *CaFRL-2* had the highest expression in the embryo (120–240 DAF), followed by the endosperm (210–240 DAF). *CaFRL-3* had the highest expression in the endosperm (120–240 DAF) and embryo (150 DAF). *CaFRL-4* had the highest expression in the perisperm (90 DAF) and embryo (150–240 DAF). Finally, *CaFRL-5* had the highest expression in the endosperm (240 DAF). As for the role of HDE in fruit development, the expression profile of *CaFRL-1* was intertwined; CaCe homeolog was prevalent in the embryo, whereas a more balanced pattern was seen in the other tissues (Fig. 6B). *CaFRL-2* differential expression had a bias toward a CaCe homeolog in all the analyzed tissues (Fig. 6B). In contrast, *CaFRL-3* did not present an expression bias, a very different pattern than that of *CaFRL-4*, for which CaCc homeolog was expressed more than the CaCe homeolog (Fig. 6B). The evaluation of *CaFRL-5* HDE in the fruits revealed an intertwined profile, with some bias toward the CaCe homeolog (Fig. 6B).

### *Coffea arabica FRL* expression during direct somatic embryogenesis

The evidence that *CaFRL*s were expressed in *C*. *arabica* embryos during fruit development prompted a hypothesis that these genes could also be expressed in “artificial” *in vitro* direct somatic embryogenesis (DSE). Detailed histological analyses of *C*. *arabica* DSE were used for evaluating the origin of early tissue embryogenesis and establish the most appropriate timing for harvesting an embryo (Fig. 4C). Eight days after inoculation, rapid cell division begins in the mesophyll, particularly in the spongy parenchyma cells (Fig. 4C). Such division intensifies 60 d after inoculation, as the first evidence of proembryogenic mass (PM) development (Fig. 4C). At this stage, mesophyll cells show an evident nucleus, dense cytoplasm, and small intercellular space. Sixty days after explant inoculation, PM appears with meristematic cells (MC) along its border. From this moment, during the different stages of development, different morphologies of the embryos (e.g., globular, heart, torpedo) begin to form (Fig. 4C).

**Figure 7:**
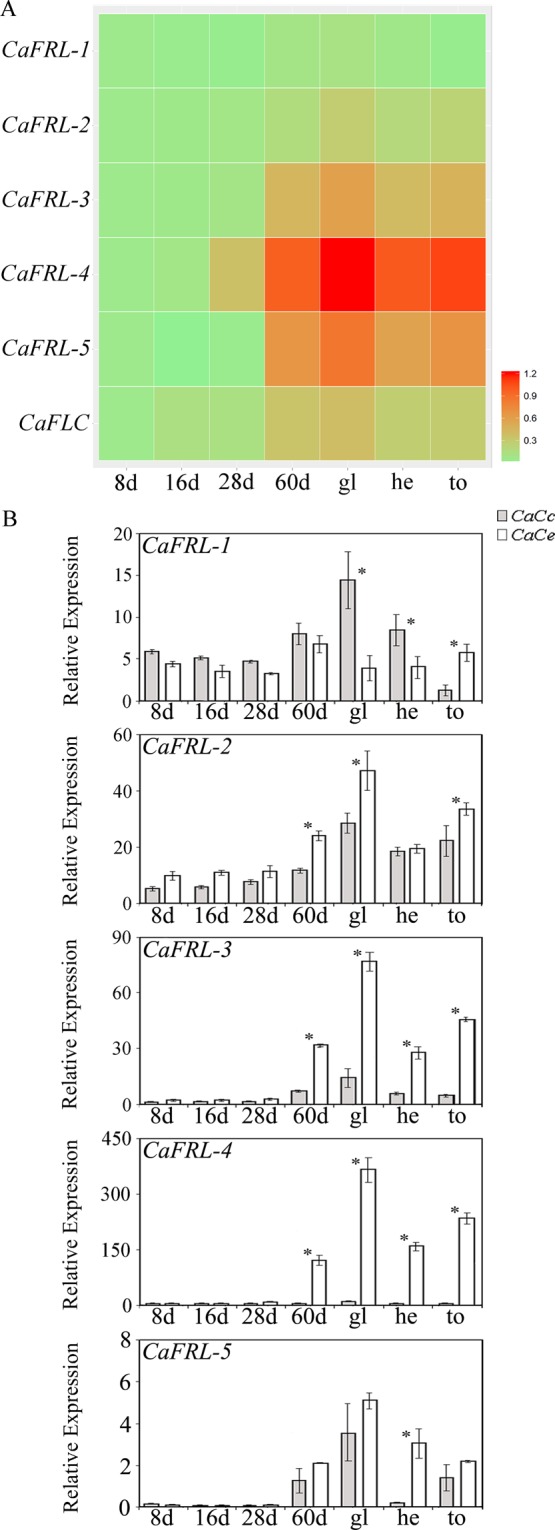
Gene expression analysis of *CaFRL* genes and *CaFLC* gene in *C*. *arabica* direct somatic embryogenesis (**A**) Heat map visualization of *CaFRL* expression in DSE at different stages. The sum of relative homeolog expressions was used as numerical input for creating the heat map scale from light green (weakly expressed) to red (strongly expressed). ‘8d’ sample was used as internal calibrator (**B**) Expression profiles of homeologous genes (CaCc and CaCe) of *CaFRL* family during DSE at different stages: 8 days (8d), 16 days (16d), 28 days (28d), 60 days (60d), globular embryos (gl), heart embryos (he) and torpedo embryos (to). Values of three technical replicates are presented as mean ± SD (error bars). Transcript abundances were normalized using the expression of *UBI* (ubiquitin) as reference gene. Asterisks indicate significant differences (P < 0.05) between homeologous genes.

Using heat-map analyses, the *CaFRL* gene expression was evaluated throughout DSE (Fig. 7). In general, all five *CaFRL* genes increased their expression at the 60-day stage, had the highest expression at the globular-embryo stage, and maintained high transcriptional levels in all the other samples of embryo shapes (Fig. 7A). In terms of HDE, *CaFRL-1* did not have an expression bias toward one subgenome (Fig. 7B). The CaCe subgenome homeolog from *CaFRL-2* appeared to be slightly more expressed than the CaCc homeolog (Fig. 7B). In the globular, heart, and torpedo stages, the *C*. *eugenioides CaFRL-3* homeolog was more expressed. The same pattern as observed for *CaFRL-4* and *CaFRL-5*, with the *C*. *eugenioides* homeolog being preferentially expressed (Fig. 7B).

### *Coffea arabica FLC* gene expression is similar to FRL gene transcription

**Figure 8:**
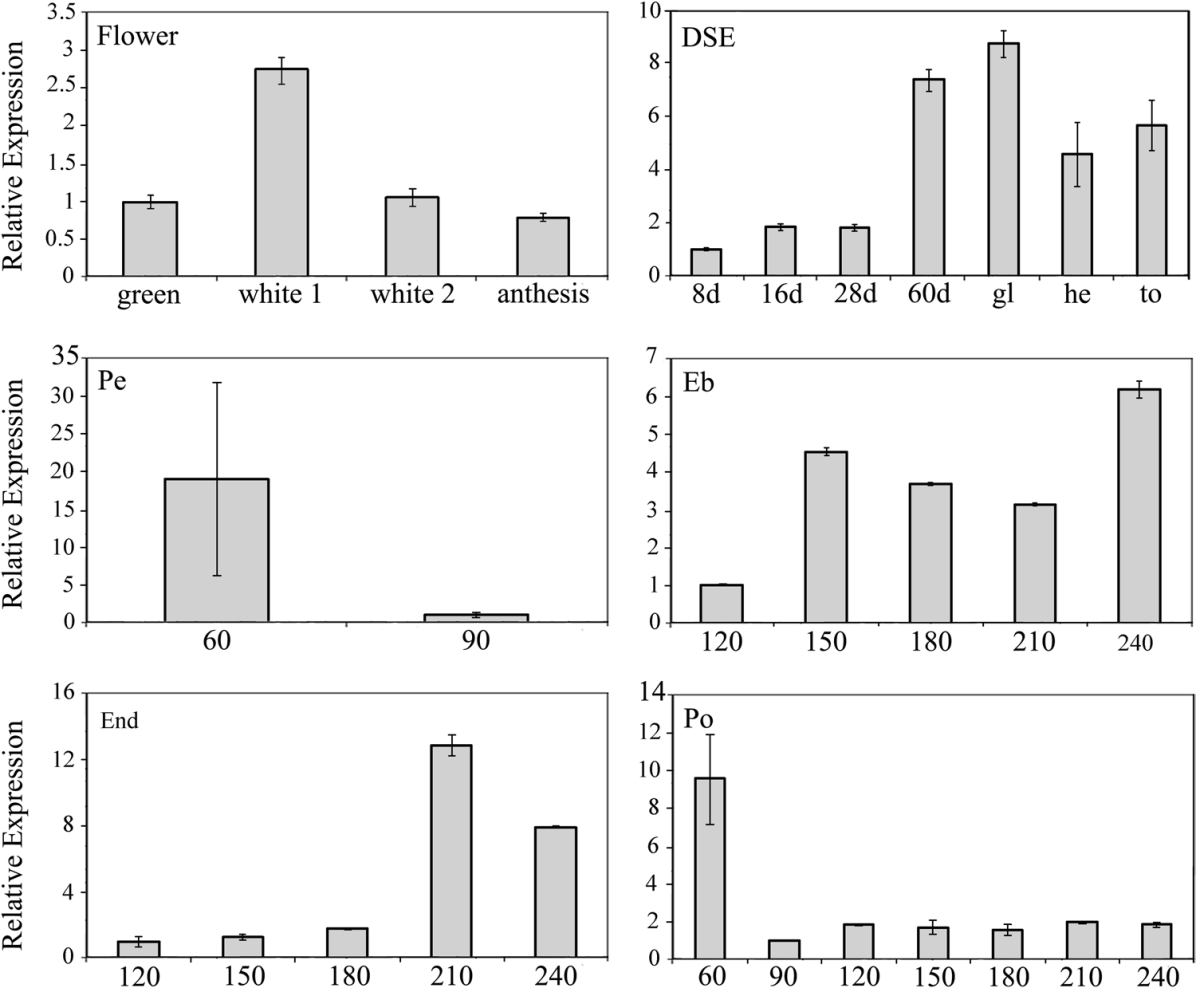
Overview of *CaFLC* gene expression. From left to right (clockwise direction): flower, direct somatic embryogenesis (DSE), fruit perisperm (Pe), embryo (Eb), endosperm (end) and fruit pericarp (po) in stages of ripening (60–240 daf, days after flowering). Values of three technical replicates are presented as mean ± SD (error bars). Transcript abundances were normalized using the expression of *UBI* (ubiquitin) as reference gene.

As mentioned above, *FRI* regulates *FLC* expression. To check whether *C*. *arabica FLC* follows *C*. *arabica FRL* genes, we examined its expression in flowers, fruits, and somatic embryos. *Arabidopsis thaliana* (FLC NP_196576.1) was used as bait for Blast analysis against *C*. *arabica*, *C*. *eugenioides*, and *C*. *canephora* genome databases. Sequences were retrieved and aligned, indicating that *C*. *arabica FLC* homeologous genes and their homeologs in C. *eugenioides* and *C*. *canephora* have very similar sequences (Supplementary Fig. S8, supplementary note). Primers designed for HDE failed to discriminate the *CaFLC* homeologs (data not shown). Using primers that aligned in both homeologous (*full* primer), *FLC* was more expressed in the floral white 1 stage (Figs 5A, 8), similar to the *FRL* genes. In fruits, *FLC* have prevalent expression in embryo and endosperm, mainly in the final stages of fruit development (Figs 6A, 8), also coinciding with *FRL* expression, especially that of *CaFRL3* and *CaFRL4*. In DSE, *CaFLC* showed the highest expression in the 60-day stage and globular embryo stage (Figs 7A, 8).

## Discussion

FRIGIDA-like proteins (FRLs) are required for regulating the flowering time in *A*. *thaliana*. In general, *Arabidopsis* accessions have two different flowering-time-related phenotypes. The first requires cold winters for flowering in spring (vernalization-responsive winter annuals), the second is a rapid-cycling summer annual. Differences in the expression of MADS-Box protein FLC, a key repressor of flowering and activator of vegetative development in *Arabidopsis*^17,27^, discriminate between the two phenotypes^28^. The *FRI* gene is known to increase the *FLC* RNA levels in winter-annual accessions, thereby delaying flowering until the *FLC* is silenced by vernalization^18,29^. In contrast, rapid-cycling accessions have low *FLC* levels because the FRI is inactive due to *FRI* allelic variation^18^. In addition, FRI forms the FRI-C complex with transcriptional activators FRIGIDA ESSENTIAL1 (FES1), FLC EXPRESSOR (FLX), and SUPPRESSOR OF FRI4 (SUF4)^30^. Moreover, the SWR complex, which acts as a chromatin remodeler to *FLC*, is recruited by FRI^20^.

Allelic sequence variation of *FRI* modulates the flowering time in *A*. *thaliana*, and *FRI* loss-of-function explains most of the variation in flowering time in early-flowering ecotypes. Nevertheless, in this study, we did not focus on the *FRI* allelic variation in *C*. *arabica* accessions, but on the homeolog variation in the species and the differential expression levels of these homeologs, particularly within reproduction-related organs. Such analyses evaluated the variation in *FRL* gene expression from an ancestry-spatiotemporal viewpoint instead of a population viewpoint, thus, connecting the *FRL* polymorphism between the *C*. *arabica* parental genomes (*C*. *canephora* and *C*. *eugenioides*) to developmental processes.

### *CaFRL* homeolog sequence analysis

Five *FRL*s were found in the *C*. *canephora* genome, and their putative orthologs were identified in the genome of *C*. *arabica* and the RNAseq assembly of *C*. *eugenioides* (Table 1). Ten *FRL*s were found in *C*. *arabica*, which was in agreement with the hypothesis that this species is an allotetraploid that is most likely derived from the hybridization of the unreduced gametes from *C*. *canephora* (or a canephoroid species group) and *C*. *eugenioides*, both apparently containing five *FRL*s. Interestingly, all the homeologs were expressed in at least one of the conditions analyzed (see below). The *C*. *arabica* and *C*. *canephora FRL* orthologs showed structural differences in the genes, including differences in gene size, number of introns, and protein size (Table 1), which could be the result of recombination, transposon action, or other molecular events during the evolution of both species.

The number of *FRL*s found in *C*. *canephora* was lower than that found in *A*. *thaliana* (8 sequences), *S*. *lycopersicum* (12 sequences), *S*. *tuberosum* (11 sequences), *V*. *vinifera* (9 sequences), *S*. *bicolor* (10 sequences), and *O*. *sativa* (11 sequences). The presence of least number of *FRLs* in *C*. *canephora* (and likely *C*. *eugenioides*) might indicate either a gene family retraction (gene loss) in these species or an expansion of *FRL*s in the other annotated species. In fact, the second hypothesis appears to be more plausible because the phylogenetic tree (Fig. 2) indicates a series of paralogs in *S*. *tuberosum*, *V*. *vinifera*, *O*. *sativa*, and *S*. *lycopersicum*. Risk *et al*.^22^ showed that Solanaceae (tomato and potato) species lack *AtFRL*-1 homeologs, and Poaceae monocots rice and sorghum do not have genes homeologous to *AtFRI* (Fig. 2), suggesting that the *FRL* sequence identity, together with the *FRL* gene family width, might be important for species-, family-, or even clade-specific developmental processes (i.e., flowering and embryogenesis) that could respond to diverse environmental adaptations.

Risk *et al*.^22^ classified *FRIGIDA*-like genes based on evident differences on the N-terminus of *A*. *thaliana* genes containing an FRI-like domain. According to the authors, the contribution of the *AtFRI* N-terminus appears to be limited to promoting *FLC* expression, whereas the C-terminus is necessary for protein-protein interactions and the promotion of consecutive *FLC* transcription. Interestingly, both CaFRLs homeologous to AtFRI (*CaFRL-3*.*1* and *CaFRL-3*.*2*) contain a C-terminus extension compared with the *A*. *thaliana* gene (Fig. 1).

### *CaFRLs* display homeologous differential expression

It was possible to discriminate homeologous genes based on the alignment among *C*. *canephora*, *C*. *eugenioides*, and *C*. *arabica FRL* sequences. This inference based on sequence alignment was confirmed by expression analysis using Taq-MAMA primer design (Fig. S3) on the leaves of the three species, which showed that CaCc *FRLs* were expressed only in *C*. *canephora* and *C*. *arabica*, and that CaCe *FRLs* were expressed only in *C*. *eugenioides* and *C*. *arabica* (Fig. 3). In addition, these results confirmed the effectiveness of this alignment-based strategy.

In general, both homeologs from each *CaFRL* gene were expressed under at least one condition in our analyses (Figs 5–7); therefore, we could not detect gene silencing in *C*. *arabica FLRs*. Instead, these results indicated a more sophisticated regulation of gene expression. This result differs from those of homeolog analyses in other allopolyploid species such as cotton, for which the genes from one subgenome have been silenced or lost during the evolution of polyploidy^31,32^. When two or more different genomes are combined within a single cell, they must respond to the consequences of genome duplication, especially with respect to duplicate copies of genes with similar or redundant functions^33^. There are some possibilities for the regulation of homeologous genes in polyploids, such as (i) retention of original or similar function for the new homeologs, (ii) functional diversification of one of the homeologs, or (iii) silencing of one of these genes^34^. However, homeologous genes could also exhibit unequal expression patterns (i.e., levels of ancestral dominance)^35^, and might vary according to different types of stress^8,36^ and among different organs^13^, as case described here. The differential expressions of these homeologs, which implicitly present sequence differences, might result in myriad combinations of protein-protein interaction that could regulate a series of developmental processes.

The presence of *cis*-elements that were connected to an environmental response (i.e., heat stress, MEJA and gibberellin response, light response; Supplementary note) is in accordance with the idea that *FRI* genes are a part of the bridge that connects environmental conditions to development. Nevertheless, there is no direct connection between *cis*-element presence/absence and gene expression of the homeologs, because most differential homeologous *cis*-elements are present in CaCe *FRL* promoters and genes from CaCc are expressed (Figs 5–7). With a more specific set of genes, the same entangled gene expression regulation described by previous authors might occur with *CaFRLs*, most likely with *trans*-factors from one subgenome acting in the other subgenome, or by epigenetic factors such as histone modification, DNA methylation, or regulatory RNAs. It is not surprising that *FRL*s could be epigenetically modulated, given that several genes involved in flowering and embryogenesis exhibit this kind of regulation^37,38^. One of the most interesting expression profiles of HDE that suggests *trans*-action was from *CaFRL-4*. During flower and fruit development, CaCc homeolog *CaFRL-4*.*1* was notably the most expressed (Figs 5, 6); however, during DSE, the expression profile changed completely with the CaCe homeolog *CaFRL-4*.*2* being more expressed (Fig. 7). One possibility is that MS medium used for DSE contains molecules that could activate the transcription of CaCe homeolog instead of CaCc. This is an example of puzzling homeologous gene regulation, which appears to rely on specific *trans-*factors from a tissue or developmental process (i.e., somatic embryogenesis vs zygotic embryogenesis; see below).

### *CaFRLs* might exert functions in late flower development

Flowering in *Coffea* plants usually occurs after a period of drought, when the onset of rain triggers flowering and anthesis. Flowering time in *Coffea* is a complex feature that is partially dependent on environmental factors, such as photoperiod and vernalization, but also on rain^39^. These external signals modulate a regulatory network to prevent the plant from blossoming too soon or too late in the season. In *Coffea*, these signals include drought, which triggers the reproductive differentiation of vegetative buds, and a rainy season, which allows flower and fruit development^39^.

A detailed morphological analysis of the *C*. *arabica* flowering mechanism had been provided by de Oliveira *et al*.^40^, indicating that, together with environmental cues, floral meristem ontogenesis is also an important factor that affects asynchronous flowering events. The same authors assessed *MADS-box* expression along floral development and discovered important differences between the spatiotemporal expression of classical *Arabidopsis MADS-box* and their orthologs in *C*. *arabica*^40^. In this sense, *MADS-box* sub- or neo-functionalization could be the cause of morphological idiosyncrasies in *C*. *arabica* flower development, such as mucilage secretion and formation of epipetalous stamens. In addition, the authors pointed out that innovative spatiotemporal coexpression of *MADS-box* (i.e., *FLC*) with its partners (i.e., *FRI*) might be related to these new functions.

Choi *et al*.^21^ reported that *Arabidopsis FLC* and *FRI* are expressed in flower buds/meristems in open flowers, and more specifically in ovules of nonvernalized plants, indicating that these genes are involved in female gametogenesis. We also found that *C*. *arabica FLC* is expressed in flowers. Barreto *et al*.^41^ detected *FLC* expression in organs exposed to abiotic and biotic stresses, We identified a quite similar expression pattern across *CaFRLs*, with high transcription at the white 1 stage and lower transcription during the later stages, except for *CaFRL-3* (*AtFRI* ortholog), the expression of which increased later during anthesis (Fig. 5A). Despite its putative importance in ovule development, it was hypothesized that during flower development, FRI might activate *FLC* to act as a repressor of *SOC1*, thus, stimulating the *SEP3* expression, and consequently, final floral organ development^42^.

### *CaFRLs* appear to be involved in embryogenesis and endosperm development

Choi *et al*.^21^ provided a comprehensive analysis of *FLC* and *FLC* regulator expression during reproductive development, including fruit development and embryogenesis. As mentioned above, the authors found that *FLC* was expressed in open flowers. Furthermore, the gene is transcribed in nonvernalized ovules, but not in pollen or vernalized ovules^21^. Nevertheless, the *FLC* expression is reactivated after fertilization in embryos but not in the endosperm. *FRI* is expressed in ovules, independent of vernalization, but not in the pollen. The gene is then reactivated in embryos following the *FLC* expression pattern^21^. C*aFLCs* have prevalent expression in embryo, similar to the *A*. *thaliana* FLC gene^21^. In our analysis, all *CaFRL*s were expressed during fruit development, although each one displaying a different expression profile (Fig. 6). Overall, the genes were expressed in the perisperm, endosperm, and embryo in diverse profiles, with much lower expression in the pericarp. One outstanding difference between *AtFRI* and *CaFRL-3* is that, although the former is conspicuously expressed only in embryos^21^, the latter is also expressed in endosperms and at a much higher level than in embryos (Fig. 6). An inspection of the expression of other *A*. *thaliana FRL*s could reveal expression patterns similar to those found in *C*. *arabica FRL*s, possibly pointing out that some *AtFRI*s are expressed in endosperm; however, differences in fruit tissue ontogenesis between *A*. *thaliana* and *C*. *arabica* can explain the discrepancy in our data.

After fecundation, *C*. *arabica* fruit contains mainly the pericarp, which is composed of the exocarp (peel), mesocarp, and endocarp, as well as perisperm, which develops from the nucleus of the ovule soon after the fertilization^43^. Perisperm is an aqueous tissue with intense cell division and expansion. At approximately 100 DAF, perisperm is progressively replaced by triploid endosperm^44^. As storage tissue, mature endosperm accumulates nutrients that are mobilized by the embryo during seed germination. The evidence that *CaFRL*s have an increased expression during the final stages of fruit development suggests that these genes could be engaged in the regulation of the physiological deposition and storage of endosperm compounds, which are quantitatively and qualitatively responsible for coffee beverage quality^45^.

### *CaFRLs* are expressed during somatic embryogenesis

One outstanding result in our data was the expression of *FRL* genes, especially that of *CaFRL-3*, *CaFRL-4*, and *CaFRL-5*, during *C*. *arabica* DSE, which is responsible for the formation of somatic embryos or embryogenic tissue directly from the explant without the development of an intermediate callus phase^46^. Interestingly, *CaFRL*s were expressed in the initial stages of embryo development during DSE, suggesting its participation in embryo maturation. Another important result was that *CaFLC* was also expressed during DSE, strongly suggesting that *FRL* genes can trigger *FLC* expression in artificial embryogenesis. Another MADS-box gene, AGL15, was found to play an essential role in somatic embryogenesis in both soybean and Arabidopsis^47^. Somatic embryogenesis (SE) is an interesting process during which plants regenerate a new plant from a single cell or a group of somatic cells^44^. Many studies have investigated the relationship between SE and zygotic embryogenesis (ZE). Nic-Can *et al*.^48^ studied SE in *C*. *canephora* and found that the genes involved in zygotic embryogenesis—*LEC1*, *BABY BOOM1*, and *WOX4*—are expressed during SE development in this plant. The fact that *FRL*s, *FLC*, and the ZE-related genes mentioned above are expressed in both ZE and SE clearly indicates that both embryogenesis processes share common developmental pathways, and thus, suggests that *FRL*s and *FLC* are embryogenesis-related genes.

By evaluating *FRL* gene expression in reproduction-related organs/tissues, we confirmed previous genome-wide homeologous gene expression pattern that indicated intertwined regulation of *C*. *arabica* homeologs. Furthermore, we found that *FRL* genes are expressed in *C*. *arabica* late flowering stages, endosperm, and embryo during ZE, and most importantly, during SE. Our study provides insights for the study of *FRL* genes, with a new perspective of FRIGIDA gene action in allopolyploids.

## Methods

### Biological material

Leaf samples were collected from *C*. *arabica* (Catuaí Amarelo IAC62), *C*. *canephora* and *C*. *eugenioides* from the germplasm of IAC (Campinas Agronomic Institute) located in Campinas, São Paulo, 54′21″S/47°03′39″W). Flowers and fruits were collected from *C*. *arabica* (Catuaí amarelo IAC62). The collection of the flowers was carried out in September 2016 according to the development of the bud flowers. The fruits were collected monthly from November 2015 to May 2016, following 60 days after flowering (DAF), 90 DAF, 120 DAF, 150 DAF, 180 DAF, 210 DAF and 240 DAF. The samples were collected in biological triplicates (Plants L7P9, L7P14 and L8P7), each plant consisting in a replica. The tissues perisperm, endosperm, pericarp and embryo were separated, frozen in liquid nitrogen and stored in a freezer at −80 °C. Direct Somatic Embryogenesis (DSE) was performed according to the methodology described by Ramos *et al*.^49^, using *C*. *arabica* leaves (Catuaí Amarelo IAC 62) as primary explant. Briefly,leaves were cut in laminar flow cabinet, removing the midrib and edges, obtaining explants of 1 cm^2^, which were inoculated with the adaxial side in contact with the culture medium, then kept in dark in a temperature of 25 °C ± 2 °C. For DSE, Murashige-Skoog (MS) medium was used with half the concentration of macronutrients and micronutrients, added with 20 g L-1 sucrose and 10 µM of isopentenyl adenine (2 iP). Samples were collected from the moment of inoculation (day 0) and throughout embryogenesis (8, 16, 28 and 60 days) until the shapes of the developing embryos at each stage could be detected (e.g., globular, heart, torpedo).

### Morphoanatomical analyses

Morphological analyses were performed from embryos obtained by Direct Somatic embryogenesis (DSE). Tissues were maintained on MS half medium, collected at the time of *in vitro* inoculation (0 days) and at different stages of development of the somatic embryos (8, 16, 28, 60 days after inoculation in the culture medium, globular embryo, heart embryo and torpedo embryo). For the anatomical analysis, the samples were fixed in FAA 50 solution (formaldehyde, acetic acid and ethanol 50%, 5: 5: 90), dehydrated in ethanol series and infiltrated in plastic resin (Leica Historesin®) according to the manufacturer’s instructions. The samples were sectioned using a manual rotary microtome (Leica®) with type C razor, in the thickness of 5 μm. Sections were stained with 0.05% toluidine blue in phosphate and citrate buffer pH 4.5 and mounted on “Entellan®” synthetic resin (Merck®). Documentation of results was performed by capturing images using the Olympus DP71 camcorder coupled to the Olympus BX 51 microscope.

### Genomic data and in silico analyses

Single-nucleotide polymorphism (SNP)–based detection of homeologous genes in *C*. *arabica* was previously described by Vidal *et al*.^3^. Biefly, the authors have used the alignment of EST sequences from *C*. *canephora* and *C*. *arabica* to infer that the sequences in *C*. *arabica* that have a SNP pattern similar to those in *C*. *canephora* originated from the CaCc subgenome, and that the ones that did not have a similar pattern were from the CaCe subgenome. These inferences were confirmed by polymerase chain reaction (PCR) using the ancestors’ DNA. Based on the expression levels, determined by counting the number of reads per tissue in each homeologous haplotype, the authors could assign genes that could hypothetically display homeologous gene expression^3^.

Identification of orthologs of the *FRIGIDA* gene family was performed using eight *Arabidopsis thaliana* FLPs as baits in BlastP searches. Their orthologs in *Coffea canephora*, *Solanum lycopersicum*, *Solanum tuberosum*, *Vitis vinifera*, *Sorghum bicolor* and *Oryza sativa* were identified in the following databases: Coffee Genome Hub^50^, NCBI (http://www.ncbi.nlm.nih.gov), TAIR (http://www.arabidopsis.org), AtGDB (http://www.plantgdb.org/AtGDB) Phytozome (http://www.phvtozome.net), Sol Genomics Network (http://solgenomics.net), SIGDB (http://www.plantgdb.org/SIGDB), Grape Genome Database (http://www.genoscope.cns.fr/externe), Gramene Database (http://www.gramene.org), and Rice Genome Annotation (http://rice.plantbiology.msu.edu). A second search was performed to identify the orthologs of the selected genes in *C*. *arabica* and *C*. *eugenioides*. The complete transcribed sequences (CDS) of the *FRL* genes of *C*. *canephora* were used as search queries in UC Davis *C*. *arabica* sequencing initiative (https://phytozome.jgi.doe.gov/pz/portal.html#!info?alias=Org_Carabica_er) and in the RNAseq reads of leaves and fruits of *C*. *eugenioides*^25^ (SRA sequence read alignment; https://www.ncbi.nlm.nih.gov/sra). Alignments were performed using the CLUSTALW tool and edited in the GeneDoc program (http://www.nrbsc.org/gfx/genedocA). Genes that did not contain specific domains were removed. Phylogenetic analysis was performed using the MEGA software^51^. The search for *cis* regulatory elements was performed using PlantCare platform (http://bioinformatics.psb.ugent.be/webtools/plantcare/html).

### RNA extraction and real-time qPCR assays

RNA was extracted using the Concert™ Plant RNA Purification Reagent (Invitrogen). RNA (1 µg) was previously treated with 1 U/µL DNAseI (Invitrogen). cDNA samples were synthesized according to according SuperScript® III Reverse Transcriptase kit protocol (Invitrogen) and used for qPCR reaction. For each reaction, 1 μl of the appropriate cDNA dilutions, 0.2 μL of the primer forward, 0.2 μL of the reverse primer at 10 mM each and 5 μL of Platinum® SYBR® Green qPCR SuperMix-UDG with ROX (Invitrogen). The reaction was supplemented with 3.6 μL Milli-Q water to a final volume of 10 μL per reaction. For each condition, the same reaction was performed three times to overlap and confirm the results in the apparatus. The data were analyzed in the program 7500 Fast Software (software v2.1.1). The samples were processed in triplicates, always accompanied by the negative controls (NTC: “in the template control”) that did not contain cDNA. The negative control in the reactions is used to verify the absence of exogenous cDNA contamination in the SYBR, primers or water mixtures. Gene expression levels were normalized to expression level of ubiquitin (UBQ10) as a constitutive reference^52^. Expression was expressed as relative quantification by applying the formula (1 + E) − ΔCt, where ΔCtartget = Cttarget gene − Ctference gene, as previously described^53^. Relative expression was The LinReg software^54^ was used to calculate the efficiency of each pair of primers per reaction. The statistical analyses (ANOVA and Tukey tests) were performed using STATISTICA software (StatSoft). The expression data was formatted by R3.4.3 software for representation. The sum of relative homeolog expressions was used as numerical input for creating the heat map. Primers were designed according to qPCR TaqMAMA method^3,26^.

## Supplementary information

Supporting Information
