# Supplementary material for: Homeologous regulation of Frigida-like genes provides insights on reproductive development and somatic embryogenesis in the allotetraploid Coffea arabica
Source: Sci Rep. 2019 Jun 11;9:8446. doi: 10.1038/s41598-019-44666-6 (PMC6560031; doi:10.1038/s41598-019-44666-6)
Supplement: Supplementary file 1 — Supporting Information [file 41598_2019_44666_MOESM1_ESM.pdf]

SUPPLEMENTARY NOTE

Homeologous regulation of Frigida-like genes provides insights about reproductive development and somatic embryogenesis in the allotetraploid *Coffea arabica*

Natalia Gomes Vieira[1,2], Ilse Fernanda Ferrari[1,2], Juliana Costa de Rezende[3], Juliana Lischka Sampaio Mayer[4], Jorge Maurício Costa Mondego [1]*

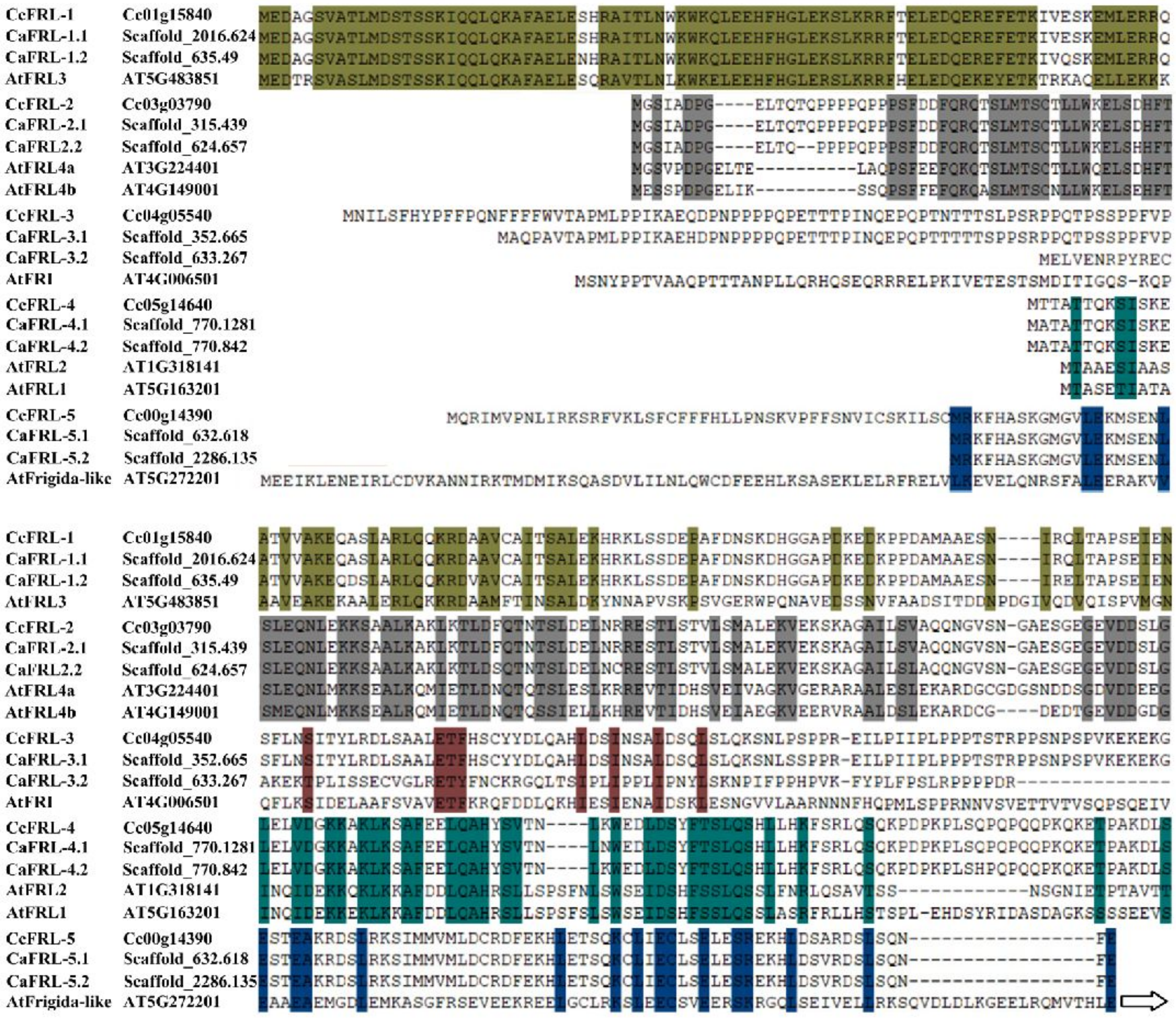

Supplementary Figure S1. N-terminal region of the Frigida-like protein families. Conserved amino acids within each family, FRL-1, FRL-2, FRL-3, FRL-4 e FRL-5 are shaded with brown, gray, red, green and blue colors, respectively. Similar amino acids are defined as acidic, basic, polar or non-polar. Family V members contain an extended N-terminal region, as indicated by the arrow. All alignments were generated using ClustalW and Gene doc software.

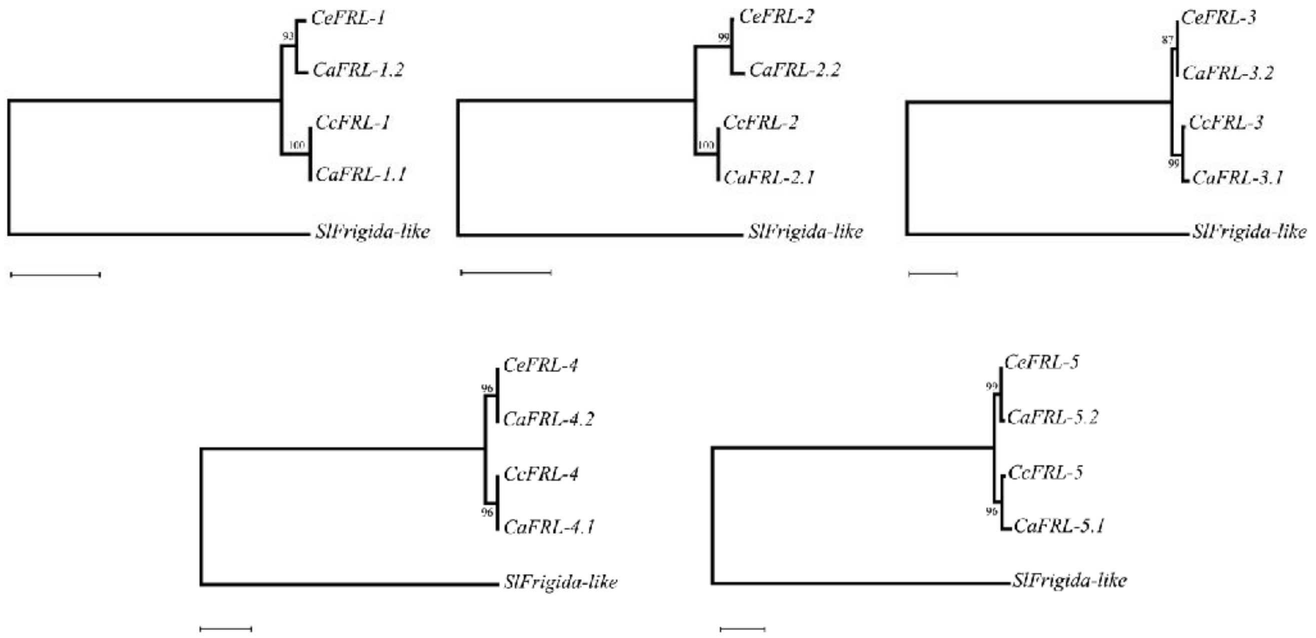

Supplementary Figure S2. Dendogram of the Frigida-like protein sequences of *C. arabica* (*CaFRL-1*, *CaFRL-2*, *CaFRL-3*, *CaFRL-4* e *CaFRL-5*), *C. canephora* (*CcFRL-1*, *CcFRL-2*, *CcFRL-3*, *CcFRL-4* and *CcFRL-5*), *C. eugenioides* (*CeFRL-1*, *CeFRL-2*, *CeFRL-3*, *CeFRL-4* e *CeFRL-5*) and *S. lycopersicum* (Sl*Frigida-like*). Notice that each FRI homeologous of *C. arabica* is positioned near *C. canephora* (FRL-x.1) or *C. eugenioides* (FRL-x.2) FRI genes. The evolutionary history was inferred using the Neighbor-Joining method conducted in MEGA7. The percentage of replicate trees in which the associated taxa clustered together in the bootstrap test (1000 replicates) is shown next to the branches. The evolutionary distances were computed using the p-distance method. Bars indicate length distance = 0.05.

Table S1. Genes and corresponding primers used for expression experiments. The primers select to expression experiments F (Forward) and R (Reverse) are indicated. The CaUBQ10*F* and *R* primers were used for the ubiquitin (UBI) as reference gene. (E) Efficiency = E + 1.

Gene Name	Target	Sequence 5'-3'	Efficiency
FRL-1	CaCc	F: CGCCGACAAGAACTCCTATCC<br>R: GTGGTTGTCAGTTGGTCCAGTG	1.91
	CaCe	F: GCCGCTGACAAGAGCTTCTATCT<br>R: GGTTGTCAGTTGGTCCAGCA	1.94
FRL-2	CaCc	F: TGACTCAGACTCAGCCTCCTCAT<br>R: CCTTCCAGAGGAGAGTGCAGC	2.05
	CaCe	F: GAGTTGACTCAGCCTCCTCGC<br>R: CCTTCCAGAGGAGAGTGCAGC	2.03
FRL-3	CaCc	F: CTGTATTGCATGGAGCTGGTTG<br>R: AACCAGTTCGCACAACACTGG	1.93
	CaCe	F: TGCATTGCATGGAGCTGGTAC<br>R: AGAACCAGTTCGCACAACACTCT	1.96
FRL-4	CaCc	F: CGCCGGACCCAGCTAAGA<br>R: AACCCGAATCATCCGCACT	2.03
	CaCe	F: CGCCGGACCCAGCTAACT<br>R: AACCCGAATCATCCGCATC	2.06
FRL-5	CaCc	F: GCAACTCCACTTCTCCTCAATGG<br>R: TTCATCTGGTGACTCAAGGTTCCT	1.93
	CaCe	F: TCAGTAACTCCACTTCTCGTCAATCT<br>R: CTTCATCTGGTGACTCAAGGTTCTC	1.84
FLC	full	F: CAGCAGGCAAGTCACGTTTTC<br>R: AACATCCACGTCGCAGAGAAC	2.03
UBI	full	F: AAGACAGCTTCAACAGAGTACAGCAT<br>R: GGCAGGACCTTGGCTGACTATA	1.98

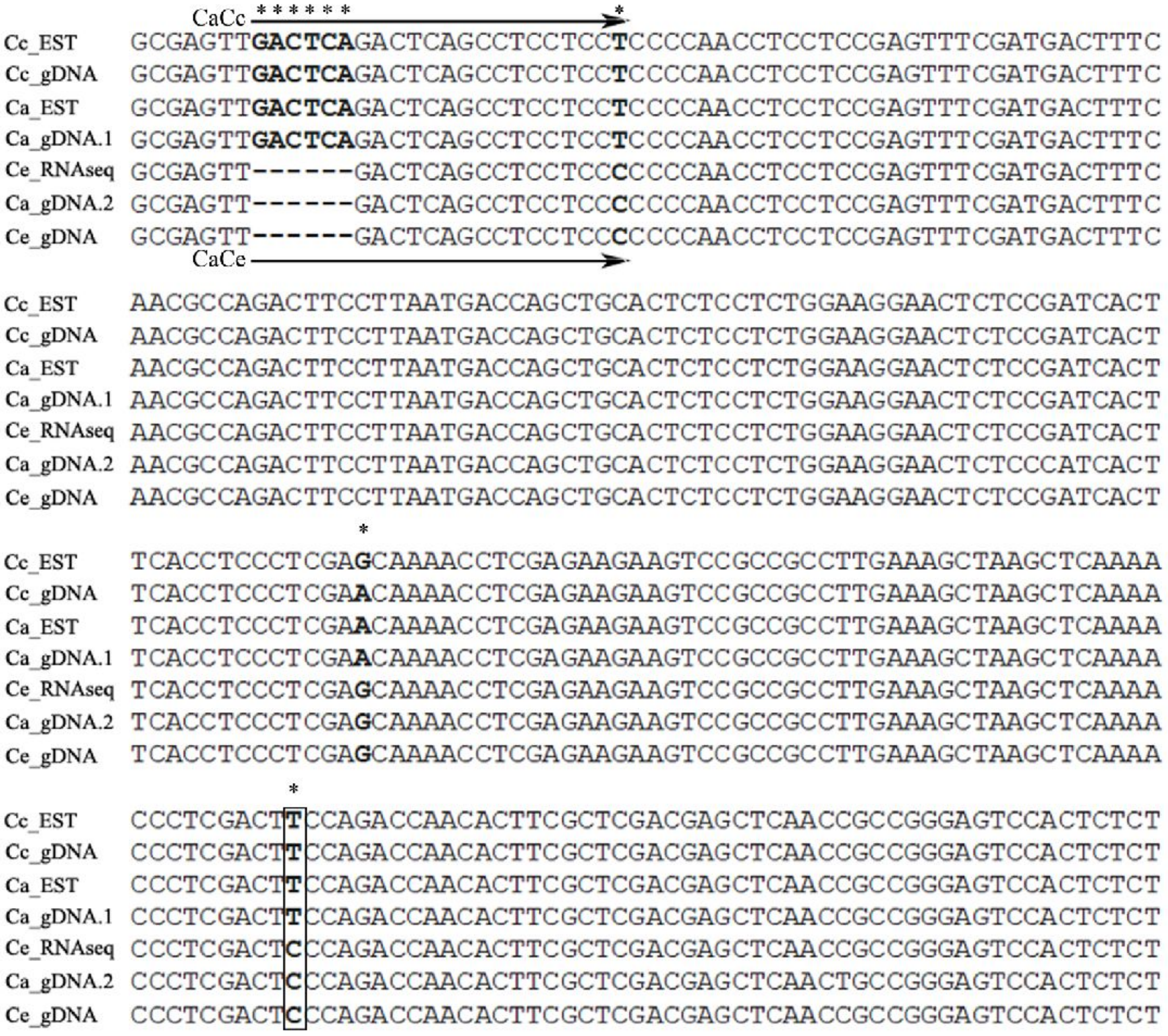

Supplementary Figure S3. Alignment of genome sequences (gDNA), Expressed Sequence Tag (EST) and RNA sequencing (RNAseq) of the *FRL-2* gene of *C. canephora* (Cc), *C. arabica* (Ca) and *C. eugenioides* (Ce). The arrows indicate homeologs *CaCc* and *CaCe*. Polymorphisms are marked with asterisks. Box shows SNPs used to design primers for TaqMAMA methodology.

Cis-elements analyses in CaFRL homologous promoters

Another approach to understanding HDE in *CaFRL* was to compare the promoter region of homeologous genes with the objective of finding putative differential *cis*-elements among those homeologs. Briefly, each *CaFRL* was localized in the *C. arabica* genome through a gene search in the Phytozome *C. arabica* databank (https://phytozome.jgi.doe.gov/pz/portal.html#!info?alias=Org_Carabica_er), and 2000 bp upstream from the start codon were selected. Promoters from homoleogous genes (e.g., *CaFRL-1.1* and *CaFRL-1.2*) were aligned and submitted to *cis*-elements *in silico* analysis in PlantCare (http://bioinformatics.psb.ugent.be/webtools/plantcare/html/). With the exception of *CaFRL-3*, all other *CaFRL* genes have differential *cis*-element between homeologous (Figs. S4–S7). The only comparison for which it was possible to find a *cis*-element that was present in a CaCc homeolog promoter but absent in a CaCe homeolog was in the *CaFRL-2* promoter that contains an HSE *cis*-element. In the other comparisons, there are *cis*-elements present in the CaCe homologous promoter and absent in the CaCc corresponding homeolog (Supplementary Figs. S4–S7; see below). For example, in the *CaFRL-1*.2 promoter, the gibberellin responsive *cis*-element (GARE), CGTCA (MeJA responsive element), and W-box (WRKY defense responsive element) were detected (Supplementary Fig. S4). Endosperm expression *cis*-element (SKN-1) and light-responsive elements CATT-box and G-Box were found in the *CaFRL-2*.2 promoter (Supplementary Fig. S5). For the *CaFRL-4*.2, I-box and GATA motif (light-responsive elements), auxin responsive element (TGA) and the MYB binding site involved in drought-inducibility (MBS) were described (Supplementary Fig. S6). Finally, *CaFRL-5*.2 promoter contained the following *cis*-elements (Supplementary Fig. S7): light-responsive element MNF1 and heat stress responsiveness (HSE).

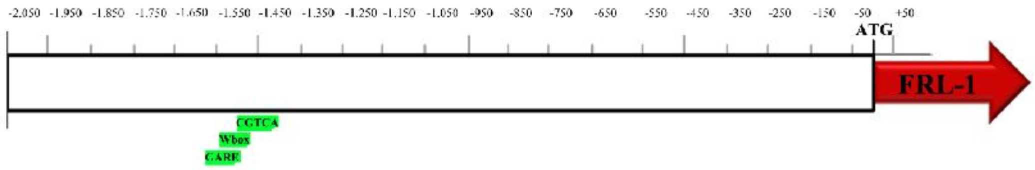

Cis-element	Function
CGTCA-motif	MeJA-responsiveness
GARE-motif	gibberellin-responsive element
W-box	defense responsive element

elements found only in CaCe promoter
elements found only in CaCe promoter

Supplementary Figure S4. Differential *cis*- elements found in *FRL-1* homeologous promoters.

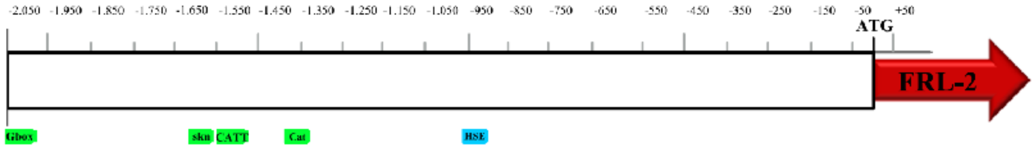

Cis-element	Function
HSE	heat stress responsiveness
Cat-box	enhancer
Skn-1_motif	endosperm expression
CATT-motif	part of a light responsive element
G-box	light responsiveness

elements found only in CaCe promoter
elements found only in CaCe promoter

Supplementary Figure S5. Differential *cis*- elements found in *FRL-2* homeologous promoters.

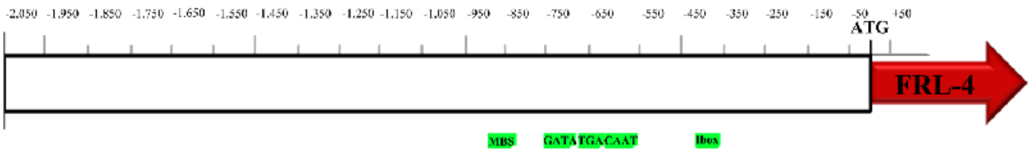

Cis-element	Function
I-box	part of a light responsive element
CAAT-box	cis-acting element in promoter and enhancer regions
GATA-motif	part of a light responsive element
TGA-element	auxin-responsive element
MBS	MYB binding site involved in drought-inducibility

elements found only in CaCe promoter
elements found only in CaCe promoter

Supplementary Figure S6. Differential *cis*- elements found in *FRL-4* homeologous promoters.

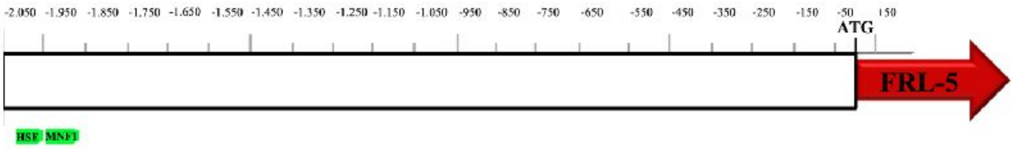

Cis-element	Function
MNF1	light responsive element
HSE	heat stress responsiveness

elements found only in CaCe promoter
elements found only in CaCe promoter

Supplementary Figure S7. Differential *cis*- elements found in *FRL-5* homeologous promoters.

Coffea arabica FLC analyis

Arabidopsis FLC protein sequences were retrieved from databank and used as baits in BLAST against *C. arabica*, *C. eugenioides* and *C. canephora* databanks. We found two sequences in *C. arabica* (XP_027097746.1 and XP_027100320.1) one in *C. eugenioides* (XP_027153948.1) and one in *C. canephora* (CDP13359.1) with identity to FLC (all have 47% of identity). The nucleotide sequences that encode those protein sequences (XM_027241945.1, XM_027244519.1, XM_027298147.1 and HG739158.1, respectively) were aligned, and we were able to discriminate the putative ancestry of *C. arabica* FLC homeologous genes. XM_027241945.1 has SNP profiles more similar to HG739158.1, while XM_027244519.1 has SNP profiles more similar to XM_027298147.1, indicating that the first C. arabica FLC is transcribed by CaCc subgenome, and the second is transcribed by CaCe subgenome (see alignment below, supplementary figure S8). Unfortunately, primers designed failed in the discrimination of *CaFLC* homeologs (data not shown).

```
HG739158.1            ------------------------------------------------------------  0
XM_027241945.1        --------TATACCGTAATATGGTAGTATTAGAGATAATGATATGTGGCTCTCAGCGGTA  52
XM_027244519.1        TAACTGCGTATACCGTAATATGGTAGTATGA---TATGTGATATGTGGCTCTCAGCGATA  57
XM_027298147.1        -----------------------------------------------------AGCGATA  7

HG739158.1            ------------------------------------------------------------  0
XM_027241945.1        ACTGGATCCCCAGGCCCTACAGTAAAATAATAATTCTGGCGTAGGAATCCTTCGACTGCC  112
XM_027244519.1        ACTGGATCCCCAGGCCCTACAGTAAAATAATAATTCTGGCGTAGGAATCCTTCGACTGCC  117
XM_027298147.1        ACTGGATCCCCAGGCCCTACAGTAAAATAATAATTCTGGCGTAGGAATCCTTCGACTGCC  67

HG739158.1            ------------------------------------------------------------  0
XM_027241945.1        GTCTTTATGAAATTCTCATTCCTTCTCCTGTGTACATATACCACTCACTCACAGCTAGGT  172
XM_027244519.1        GTCTTTATGAAATTCTCATTCCTTCTCCTCTGTACATATACCACTCACTCACAGCTAGGT  177
XM_027298147.1        GTCTTTATGAAATTCTCATTCCTTCTCCTCTGTACATATACCACTCACTCACAGCTAGGT  127

HG739158.1            ------------------------------------------------------------  0
XM_027241945.1        AGAAACAAATACAGGTTCAAATTCCGAATCTCGGAGAATTAGGGCAAAAACCAGAACCAA  232
XM_027244519.1        AGAAACGAATACAGGTTCAAATTCCGAATCTCGGAGAATTAGGGCAAAAACCAGAACCAA  237
XM_027298147.1        AGAAACGAATACAGGTTCAAATTCCGAATCTCGGAGAATTAGGGCAAAAACCAGAACCAA  187

HG739158.1            ------------------------------------------------------------  0
XM_027241945.1        CGATCTGTTTTTTCTTATCTGCCAGTTCGTACCTTTTTT-TTTTCTTTTTGGGGGGAGCG  291
XM_027244519.1        CGATCTGTTTTTTCTTATCTCCCAGTTCGTACCTTTTTTTTTTTCTTTTTGGGGGGAGCG  297
XM_027298147.1        CGATCTGTTTTTTCTTATCTCCCAGTTCGTACCTTTTTT-TTTTCTTTTTGGGGGGAGCG  246

HG739158.1            -----------------------ATGGGGCGGAGGAAGGTGGAGATTAAGAAAATCGAGG  37
XM_027241945.1        GGGTACCGACGAAGGAATTAAGGATGGGGCGGAGGAAGGTGGAGATTAAGAAAATCGAGG  351
XM_027244519.1        GGGTACCGACGAAGGAATTAAGGATGGGGCGGAGGAAGGTGGAGATTAAGAAAATCGAGG  357
XM_027298147.1        GGGTACCGACGAAGGAATTAAGGATGGGGCGGAGGAAGGTGGAGATTAAGAAAATCGAGG  306
                                             *************************************

HG739158.1            ACAAGAACAGCAGGCAAGTCACGTTTTCCAAGCGGAGAAGCGGACTGATGAAGAAAGCCA  97
XM_027241945.1        ACAAGAACAGCAGGCAAGTCACGTTTTCCAAGCGGAGAAGCGGACTGATGAAGAAAGCCA  411
XM_027244519.1        ACAAGAACAGCAGGCAAGTCACGTTTTCCAAGCGGAGAAGCGGACTGATGAAGAAAGCCA  417
XM_027298147.1        ACAAGAACAGCAGGCAAGTCACGTTTTCCAAGCGGAGAAGCGGACTGATGAAGAAAGCCA  366
                      ************************************************************

HG739158.1            AGGAACTTTCCGTTCTCTGCGACGTGGATGTTGCTGTCCTCATCTTCTCTGGTCGCGGCA  157
XM_027241945.1        AGGAACTTTCCGTTCTCTGCGACGTGGATGTTGCTGTCCTCATCTTCTCTGGTCGCGGCA  471
XM_027244519.1        AGGAACTTTCCGTTCTCTGCGACGTGGATGTTGCTGTCCTCATCTTCTCTGGTCGCGGCA  477
XM_027298147.1        AGGAACTTTCCGTTCTCTGCGACGTGGATGTTGCTGTCCTCATCTTCTCTGGTCGCGGCA  426
                      ************************************************************

HG739158.1            AGCTCTACGACTTCTGCAGCACCAACAGTTTGGCCAAGATCCTACAACGATATCACAACT  217
XM_027241945.1        AGCTCTACGACTTCTGCAGCACCAACAGTTTGGCCAAGATCCTACAACGATATCACAACT  531
XM_027244519.1        AGCTCTACGACTTCTGCAGCACCAACAGTTTGGCCAAGATCCTACAACGATATCGCAACT  537
XM_027298147.1        AGCTCTACGACTTCTGCAGCACCAACAGTTTGGCCAAGATCCTACAACGATATCGCAACT  486
                      ******************************************************.*****

HG739158.1            ATGCAGAAGCAGAAGACGGGCCTGCAAGAATTAGCGGTGTAGAGAAACGTAACCCTGAAG  277
XM_027241945.1        ATGCAGAAGCAGAAGACGGGCCTGCAAGAATTAGCGGCGTAGAGAAACGTAACCCTGAAG  591
XM_027244519.1        ACGCAGAAGCAGAAGACGGGTCTGCAAGAATTAGCGGCGTAGAGAAACGTAACCCTGAAG  597
XM_027298147.1        ACGCAGAAGCAGAAGACGGGTCTGCAAGAATTAGCGGCGTAGAGAAACGTAACCCTGAAG  546
                      * ****************** **************** **********************

HG739158.1            GCAGAAATGTCGTGACAATCAGAAAGCTGCTGGAAAAAAATCTAATGTTGTCTCTGTTTT  337
XM_027241945.1        GCAGAAATGTCGTGACAATCAGAAAGCTGCTGGAAAAAGTT-------------------  632
XM_027244519.1        GCAGAAATGTCGTGACAATCAGAAAGCTGCTGGAAAAAGTT-------------------  638
XM_027298147.1        GCAGAAATGTCGTGACAATCAGAAAGCTGCTGGAAAAAGTT-------------------  587
                      **************************************.:*

HG739158.1            TCAACAGGGATCTAGAGGAGCCAGATGTTGACCACCTTAACCTGAGTGAACTAGTGCAAT  397
XM_027241945.1        --GAAAGGGATCTAGAGGAGCCAGATGTTGACCACCTTAACCTGAGTGAACTAGTGCAAT  690
XM_027244519.1        --GAAAGGGATCTAGAGGAGCCAGATGTTGACCACCTTAACCTGAGTGAACTAGTGCAAT  696
XM_027298147.1        --GAAAGGGATCTAGAGGAGCCAGATGTTGACCACCTTAACCTGAGTGAACTAGTGCAAT  645
                        .*.*******************************************************

HG739158.1            TGGAAGAACAACTTGAAGATGCACTCATTCAAACAAGATCTAGGAAGACACGATTACTGA  457
XM_027241945.1        TGGAAGAACAACTTGAAGATGCACTCATTCAAACAAGATCTAGGAAGACACGATTACTGA  750
XM_027244519.1        TGGAAGAACAACTTGAAGATGCACTCATTCAAACAAGATCTAGGAAGACACGATTACTGA  756
XM_027298147.1        TGGAAGAACAACTTGAAGATGCACTCATTCAAACAAGATCTAGGAAGACACGATTACTGA  705
                      ************************************************************
```

```
HG739158.1          TGGAATCAATAACCAGTCTAAGTGAAGTGGAAAAGATGCTGAGGGAAGAAAACAAGCTTC  517
XM_027241945.1      TGGAATCAATAACCAGTCTAAGTGAAGTGGAAAAGATGCTGAGGGAAGAAAACAAGCTTC  810
XM_027244519.1      TGGAATCAATAACCAGTCTAAGTGAAGTGGAAAAGATGCTGAGGGAAGAAAACAAGCTTC  816
XM_027298147.1      TGGAATCAATAACCAGTCTAAGTGAAGTGGAAAAGATGCTGAGGGAAGAAAACAAACTTC  765
                    *******************************************************.****

HG739158.1          TGCAAAATAAGGTAGCTGCAGGTACACCCAATGAGAAGAGGAATGACTTGATCCTTGAAT  577
XM_027241945.1      TGCAAAATAAGGTAGCTGCAGGTACACCCAATGAGAAGAGGAATGACTTGATCCTTGAAT  870
XM_027244519.1      TGCAAAATAAGGTAGCTGCAGGTACATCCAATGAGAAGAGGAATGACTTGATCCTTGAAT  876
XM_027298147.1      TGCAAAATAAGGTAGCTGCAGGTACATCCAATGAGAAGAGGAATGACTTGATCCTTGAAT  825
                    ************************** *********************************

HG739158.1          TTGGGGACCTTACACACGTTGGAATGATTTCTGGGCAGCGACAGGCTATGCTTGAACTAC  637
XM_027241945.1      TTGGGGACCTTACACACGTTGGAATGATTTCTGGGCAGCGACAGGCTATGCTTGAACTAC  930
XM_027244519.1      TTGGGGACCTTACACACGTTGGAATGATTTCTGGGCAGCGACAGGCTATGCTTGAACTAC  936
XM_027298147.1      TTGGGGACCTTACACACGTTGGAATGATTTCTGGGCAGCGACAGGCTATGCTTGAACTAC  885
                    ************************************************************

HG739158.1          TTTAA-------------------------------------------------------  642
XM_027241945.1      TTTAAGAGTAGAAGATAGTGAGACAAGCCAGTTTGTGCCTTTCTGAGTGCTCCAAGGGAA  990
XM_027244519.1      TTTAAGAGTAGAAGATAGTGAGACAAGCCAGTTTGTGCCTTTCTGAGTGCTCCAAGGGAA  996
XM_027298147.1      TTTAAGAGTAGAAGATAGTGAGACAAGCCAGTTTGTGCCTTTCTGAGTGCTCCAAGGGAA  945
                    *****

HG739158.1          ------------------------------------------------------------  642
XM_027241945.1      AATATACCCGTTAAGGCAATGTGATCCTACTATACAACTCTTCATAAGCAACTTTTTATA  1050
XM_027244519.1      AATATACCCGTTAAGGCAATGTGATCCTACTATACAACTCTTCATAAGCAACTTTTTATA  1056
XM_027298147.1      AATATACCCGTTAAGGCAATGTGATCCTACTATACAACTCTTCATAAGCAACTTTTTATA  1005

HG739158.1          ------------------------------------------------------------  642
XM_027241945.1      ACTTGGTTTTGCGTCTGACATCACTCCGCTTCATAACATGAGATTGAAGTGGGGTACGTT  1110
XM_027244519.1      ACTTGGTTTTGCGTCTGACATCACTCCGCTTCATAACATGAGATTGAAGTGGGGTACGTT  1116
XM_027298147.1      ACTTGGTTTTGCGTCTGACATCACTCCGCTTCATAACATGAGATTGAAGTGGGGTACGTT  1065

HG739158.1          ------------------------------------------------------------  642
XM_027241945.1      CTCCACTTGCATTGTTCTGTCACATGCTAACCCAGGTTTCTGATTAAGCAAAAGAGAATG  1170
XM_027244519.1      CTCCACTTGCATTGTTCTGTCACATGCTAACCCAGGTTTCTGATTAAGCAAAAGAGAATG  1176
XM_027298147.1      CTCCACTTGCATTGTTCTGTCACATGCTAACCCAGGTTTCTGATTAAGCAAAAGAGAATG  1125

HG739158.1          ------------------------------------------------------------  642
XM_027241945.1      AATCTTAAAGCAAAGCAGGTTGCCTGCAAGTTGGTCA-----------------------  1207
XM_027244519.1      AATCTTAAAGCAAAGCAGGTTGCCTGCAAGTTGGTCA-----------------------  1213
XM_027298147.1      AATCTTAAAGCAAAGCAGGTTGCCTGCAAGTTGGTCATTGGTGAGTATTCTAACTAAAAA  1185

HG739158.1          ------------------------------------------------------------  642
XM_027241945.1      ------------------------------------------------------------  1207
XM_027244519.1      ------------------------------------------------------------  1213
XM_027298147.1      ATACTTAAAACTGCTTGTACCACCTCAATGGCATACAGACAAATTTGGTAATGTAATTAC  1245
```

Supplementary figure S8: Alignment of Coffea *FLC* gene sequences from *C. arabica* (XM_027241945.1 and XM_027244519.1), *C. canephora* (HG739158.1) and *C. eugenioides* (XM_027298147.1). In green: start codon, In red: stop codon, In yellow: SNP position at which one *C. arabica* homeologous sequence is identical to *C. canephora,* In blue: SNP position at which one *C. arabica* homeologous sequence is identical to *C. eugenioides.*
